# AI-driven reconstruction of the evidence architecture of hydrogel-based intervertebral disc repair research

**DOI:** 10.3389/fcell.2026.1860034

**Published:** 2026-05-15

**Authors:** Yifan Wang, Junyao Cheng, Chuyue Zhang, Taoxu Yan, Zheng Tian, Jianheng Liu, Qinghan Li, Zheng Wang

**Affiliations:** 1 Department of Orthopaedics, Chinese PLA General Hospital, Beijing, China; 2 Department of Xinmin Orthopedic, China-Japan Union Hospital of Jilin University, Changchun, China

**Keywords:** artificial intelligence, evidence architecture, hydrogel, intervertebral disc degeneration, thematic maturity

## Abstract

**Background:**

Intervertebral disc degeneration (IVDD), the leading cause of chronic low back pain, imposes heavy socioeconomic burdens, and conventional treatments cannot achieve biological disc repair. Hydrogels are promising for IVDD regeneration, but traditional bibliometrics fails to uncover the field’s evidence architecture, thematic maturity and structural bottlenecks.

**Methods:**

We retrieved 1,085 English publications (2016–2025) from three major databases, and developed an AI-driven framework to reconstruct the field’s evidence hierarchy *via* a five-level experimental-stage-based classification and five-dimensional thematic maturity assessment.

**Results:**

The field has entered the mid-to-late preclinical stage, dominated by Level 3 (animal/*ex vivo*) evidence, with only 7 Level 5 (advanced translational) studies. Research focus has shifted to Level 4 (degeneration/mechanism) validation. Three mature pillars were identified: anti-inflammation/microenvironment regulation, ECM biomimetic hydrogels and nucleus pulposus regeneration. Annulus fibrosus repair is the critical bottleneck, while mechanical functional restoration and composite/multifunctional hydrogels are the most promising emerging directions.

**Conclusion:**

This AI-assisted evidence reconstruction maps the unbalanced transition of the field from biomaterial-centered exploration to degeneration-context integration and functional restoration. Future progress will rely on deeper structural, mechanistic and functional integration rather than expanding isolated material platforms, providing guidance for research prioritization and clinical translation.

## 1 Introduction

Intervertebral disc degeneration (IVDD) is the main cause of long-term low back pain, and it is not easy to realize biological repair. The structure of intervertebral disc is complex, and its biological environment is very limited-poor blood supply, weak self-repair ability and harsh local conditions. It is these characteristics that make the therapeutic methods based on biomaterials become more and more promising research directions for scientists (Knezevic et al., 2021; Lee et al., 2025; Zheng et al., 2025; Latka et al., 2026).

Hydrogels have become a hot field in the research of the restoration of the intervertebral disc, because they have great characteristics: high water content, like the extracellular matrix in our body, which can be injected, and their physical and chemical properties can be adjusted, and they can cooperate well with cells and bioactive agents. With the passage of time, the method based on hydrogel has developed from a simple scaffold-like structure to a more advanced regenerative framework. Biomimetic matrix design is added to these new frameworks, which can protect nucleus pulposus and transport stem cell. Extracellular vesicles, controlled release system, adjustment of inflammation and technologies focusing on mechanical recovery are also used (Correa et al., 2021; Zhang et al., 2021; Dong et al., 2025). Despite the rapid development of this field, we still do not know how the internal evidence framework is organized. Some current reviews and literature analysis may tell us what articles we like to publish and which topics often appear, but they can’t help us to judge whether a research method represents a solid research foundation, a platform with active technology but loose contact, or a fundamental problem that has not been solved. Nowadays, the directions of biomaterials, degeneration biology and functional restoration are more and more closely combined, and it is impossible to really understand which step this field has developed by counting the number of papers (Chen et al., 2023; Wang et al., 2024; Zhao et al., 2024).

**FIGURE 1:**
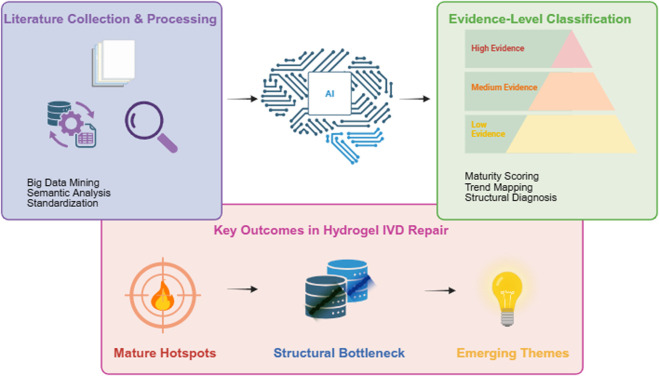
AI-centered conceptual framework for evidence architecture reconstruction in hydrogel-based intervertebral disc repair research.

Artificial intelligence (AI) provides us with a super powerful way to solve this problem. It does not regard all kinds of papers as a bunch of unrelated articles as we usually do, but it can use some clever techniques, such as unifying the meaning of words, grading evidence, and finding out the theme structure of articles, to turn all kinds of data into an organized evidence framework (Shi et al., 2023; Yang Z. et al., 2023; Dai et al., 2025). In this way, AI will not simply process words; It has become a core analysis center, connecting the steps of collecting and preliminarily processing documents, classifying evidence and finally drawing important conclusions. The overall analytical logic is summarized in Figure 1, where literature collection and processing are connected to AI-driven evidence classification and subsequent structural interpretation, ultimately leading to three key outputs: mature research hotspots, structural bottlenecks, and emerging themes with high integration potential.

In order to do this, this study designed an AI-driven analysis toolkit to sort out the evidence distribution in the research of Hydrogel-based interdisciplinary disc repair. We organize the existing literature into a clear framework through a whole set of processes, including corpus normalization, evidence ST comparison and thematic maturity evaluation, so that we can accurately find out the existing research frontiers, structural gaps and emerging interdisciplinary topics. In this way, our research not only wants to describe the development track of this field, but also wants to show how its evidence hierarchy and thematic abstraction have evolved over time.

## 2 Materials and methods

### 2.1 Data sources and corpus construction

We found many academic articles about repairing intervertebral disc with hydrogel, which came from three main academic databases: Web of Science Core Collection, Scopus, and PubMed. We only studied English articles published in the decade from 2016 to 2025. Our search method is mainly to see if hydrogels are mentioned, and words related to intervertebral disc, such as disc degeneration, nucleus pulposus, annulus fibrosus, and repair/regeneration. In the process of sorting out the articles, we have removed all the researches on intervertebral disc that have nothing to do with hydrogel methods. After we combined the results of the three databases, removed the duplicate articles, and took time to filter them, we finally got 1,085 articles, ready for in-depth research.

### 2.2 Metadata harmonization and deduplication

In order to make all databases consistent, we have created a unified framework to standardize metadata, which contains a lot of important information, such as title, authors, abstract, keywords, publication year, journal name, DOI, institutional affiliations, document type and originating database. We use a step-by-step method to get rid of duplicate entries: first, we make accurate matching according to DOI; The second step is to accurately match the records without DOI according to the title; Finally, use fuzzy title matching carefully, and ensure that the publication years are consistent. When we find duplication between different databases, we will keep the record with the most complete information as the main entry, and then add the unique metadata in other records. In the end, we only kept the publications from 2016 to 2025.

### 2.3 Terminology normalization and AI-ready corpus preparation

In order to eliminate the confusion caused by the inconsistency of terms in different databases and studies, we thoroughly cleaned up and standardized the titles, abstracts and keywords before using AI analysis. Our text processing process is comprehensive: unifying Unicode encoding, sorting out spaces, deleting text fragments in URL and DOI format, converting all contents into lowercase, and unifying symbol formats. Then, we specially customized a terminology comparison table for the study of intervertebral disc, so as to unify the related synonyms such as biomaterials, anatomical sites, degradation, bioactive substances and molecular results. In order to simplify the terminology, we have unified the format of various references to gelatin method, transverse disc degeneration, extracellular vesicles, nucleus pulposus and annulus fibrosus. After doing this, we built a database that can be used by AI with a sorted title, abstract and standard keywords, so that we can have unified written materials for evidence classification and subject analysis. At the same time, we also developed a framework that can identify entities, which is convenient for later identifying terms related to materials, mechanisms, disease background and results.

### 2.4 AI-assisted evidence-level classification

In order to find out what the articles about hydrogel repairing intervertebral disc said, we designed an AI-assisted method to classify each study into one of five grades according to the existing rules. This is not to see how many times the articles have been cited or how powerful the magazines are, but to classify them according to the most advanced experimental or application results they have actually achieved.

Level 1 includes the basic design and physical and chemical analysis of materials, and studies their rheological behavior, viscoelasticity, expansion characteristics, degradation mode, injectability, gel behavior, porosity, crosslinking density and other related material properties. Level 2 focuses on *in vitro* biological evaluation, testing cell health, growth rate, adhesion ability, exercise mode, extracellular matrix production, cartilage maturity, and experimental settings using nucleus pulposus cells, annulus fibrosus cells or various stem cell groups. Level 3 involves animal tissue or *in vitro* verification research, including puncture model, rodent tail or rabbit intervertebral disc system, *in vitro* intervertebral disc culture, histological examination and imaging technology to evaluate the progress of tissue repair. Level 4 verifies the degenerative environment or disease mechanism, which requires that there must be both degeneration-related terms and pathological indicators-for example, the description of IVDD should appear together with inflammatory process, apoptosis, aging markers, oxidative stress response, MMP and ADAMTS expression, macrophage activity or immune regulation signals. Level 5 stands for advanced transformation research or long-term functional integration. This level is only reserved for those studies that meet more stringent combination criteria, such as large-scale animal model terminology, long follow-up period, and/or evidence of functional recovery-this includes biomechanical recovery or continuous functional regeneration results.

The title, abstract and standardized keywords of each article are carefully checked according to different levels of standards. Because some studies may contain multiple levels of content, the final classification is determined according to the highest level of evidence that the study can achieve. This grading method focuses on the most advanced experimental stage of each paper, not its initial steps. Those studies that do not meet any grading standards are classified as unclassified.

### 2.5 AI-driven thematic assignment and thematic maturity assessment

In order to get rid of the traditional keyword matching analysis, we developed an AI-driven topic classification system with rules. This system can correspond each paper to one or more easily understood research topics. This labeling process is to let the AI-optimized document library match our pre-determined thematic rule sets that are unique to each field. The final theme framework includes: Injectable Hydrogels, ECM Biomimetic Hydrogels, Stem Cell Delivery, Exosome or EV Delivery, NP Regeneration, AF Repair, Anti-infiltration and IVDD microenvironment, mechanical functional restoration, controlled release and bioactive cargo, and composite or multifunctional hydrogels, *etc.* Because many studies often involve multiple scientific aspects, we allow an article to have multiple topics. On the basis of these document-level topic assignments, we then use five key indicators to evaluate the maturity of each topic: the first is “frequency”, which is to calculate the total number of papers related to a specific topic. The average evidence level represents the average evidence score of papers published under each topic, while the recent increase ratio indicates the proportion of papers related to this topic in the recent period. The coverage rate of disease background measures the percentage of subject papers containing degradation-related terms, while the coverage rate of function quantifies the proportion of papers containing functional recovery-related terms. In order to calculate these coverage indicators, we re-examined the articles assigned to each topic using special disease background and functional terminology. Finally, according to the development track, each topic is classified into one of four structural categories: mature hotspot, structural bottleneck, high-integration emerging theme or intermediate/mixed theme. Mature hot spots refer to those topics that are often discussed, which are supported by many practical studies and closely related to the disease background. The structural bottleneck refers to those topics that lack strong evidence and fail to establish meaningful practical connections, although there are many relevant documents. Emerging topics with high integration are defined as moderately popular directions with rapidly increasing attention, more evidence support and closer integration with the disease framework. Those topics that do not quite meet the above classification are marked as intermediate or mixed. This method turns topic identification into a systematic maturity assessment, which enables us to clearly distinguish which are solid research foundations, which are areas that need to be broken through, and which are directions with great development potential in the future.

## 3 Results

### 3.1 Overall analytical framework and evidence-level architecture

**FIGURE 2:**
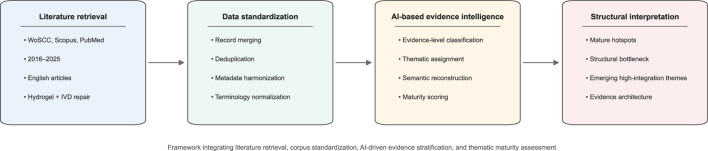
AI-driven analytical framework for evidence architecture reconstruction in hydrogel-based intervertebral disc repair research.

Figure 2 shows a comprehensive and AI-driven analysis method we used in this study. By integrating literature search from multiple databases, standardizing our literature, classifying evidence according to quality by AI, and evaluating the development of the subject, our workflow has successfully turned a pile of scattered references into a well-organized knowledge framework. This framework helps us to clarify the evidence structure in hydrogel disc repair research.

**FIGURE 3:**
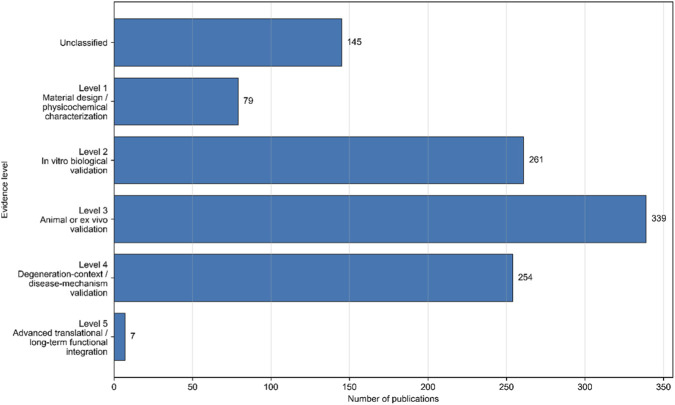
Overall evidence-level composition of hydrogel-based intervertebral disc repair research (2016–2025).

After integrating the database, eliminating duplication and narrowing the time range to 2016–2025, we finally got 1,085 studies, which can be used for subsequent analysis. As shown in Figure 3, the overall level of evidence shows that this field is mainly dominated by Level 3 (animal or *in vitro* verification), followed by Level 2 (*in vitro* biological verification) and Level 4 (verification of degraded environment or disease mechanism), while Level 5 (advanced transformation or long-term functional integration) research is still rare. This trend shows that the restoration science of IVD based on hydrogel has got rid of the exploration of pure materials, but it is not fully mature in advanced transformation and long-term functional reconstruction.

**FIGURE 4:**
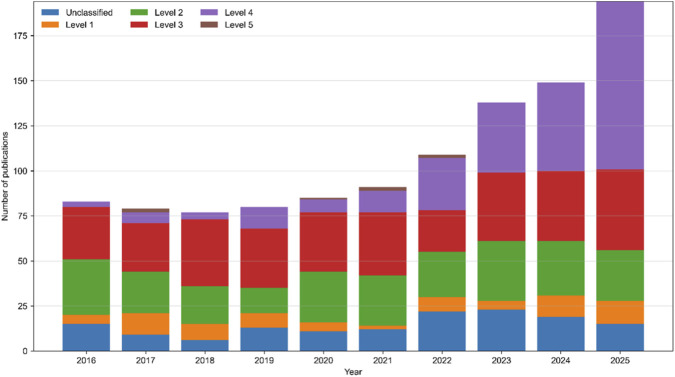
Temporal evolution of evidence levels in hydrogel-based intervertebral disc repair research (2016–2025).

Figure 4 shows more clearly how the pattern of evidence changes over time. From 2016 to 2025, the number of academic papers has soared every year, and the basis of evidence has also undergone major changes. In the early stage, this field mainly relied on the research of Level 2 and Level 3. By the end of the research period, it was Level 4 research that really grabbed the limelight, and they grew the fastest. This trend shows that contemporary research pays more and more attention to degradation-environment integration, inflammatory microenvironment regulation and disease mechanism-oriented verification, and is no longer limited to traditional material characterization or separate biological feasibility assessment. Although there have been various limitations in the whole research process, the research on Level 5 is still very few, which shows that truly advanced transformation research and long-term functional recovery are still a big problem in this field. On the whole, these findings show that the research of intervertebral disc repair based on hydrogel is in the pre-clinical, middle and late stage: this discipline has definitely surpassed the initial research on material properties, and the verification of diseases has been paid more and more attention, but the high-level transformation application still falls short of expectations.

### 3.2 Thematic landscape of hydrogel-based IVD repair research

**FIGURE 5:**
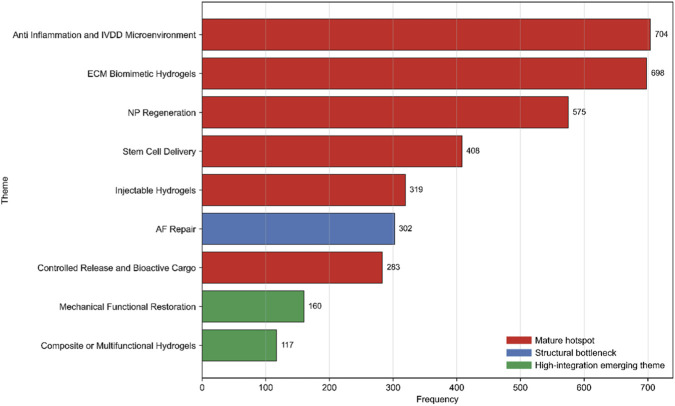
Thematic frequency landscape of hydrogel-based intervertebral disc repair research.

In order to know more clearly what is inside this research field, we have carefully looked at its themes. As shown in Figure 5, this theme layout is obviously supported by several important research directions, such as anti-infection and ivdd microenvironment, ECM Biomimetic Hydrogels, and NP Regeneration. In addition, we also pay special attention to Stem Cell Delivery, Injectable Hydrogels, and controlled release and bioactive cargo. These findings show that there are three main pillars in this field: one is to design bionic materials, the other is to find ways to regenerate NP (nucleus pulposus), and the other is to study how to improve the environment that makes it worse.

Among the research topics, Anti-Inflammation and IVDD Microenvironment are the most popular directions, which shows that people are paying more and more attention to microenvironment regulation and pathophysiological targeted therapy. Similarly, ECM Biomimetic Hydrogels and NP Regeneration are also the key areas of research.

Protecting and restoring teeth has always been the most important foundation in this discipline. At the same time, there are a lot of papers published in research fields such as stem cell transplantation, injectable biomaterials and controlled delivery systems with bioactive agents. This shows that those platform technologies and methods that focus on effective delivery of substances are becoming more and more advanced and complex.

### 3.3 Thematic maturity identifies backbones, bottlenecks, and integrations

**FIGURE 6:**
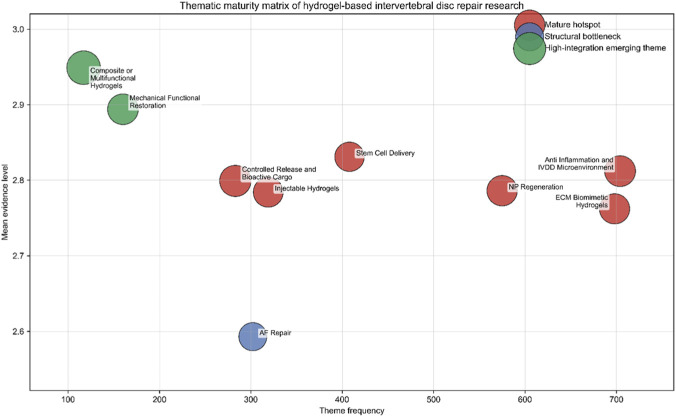
Thematic maturity matrix of hydrogel-based intervertebral disc repair research.

In order to not only simply count the frequency of research topics, but also combine the number of publications, the average strength of evidence and the recent growth trend, and draw a topic maturity map in this field. As Figure 6 shows, the development of this field does not go hand in hand with all themes, but is divided into three distinct parts: mature hot areas, structural bottlenecks, and highly integrated emerging directions. The first category is mature hotspots, such as Anti-Inflammation and IVDD Microenvironment, ECM Biomimetic Hydrogels, NP Regeneration, and some platform-driven mature research directions, such as Stem Cell Delivery, Injectable Hydrogels, controlled release and bioactive cargo. When it comes to repairing intervertebral discs with hydrogels, these research topics are particularly hot, because they are not only published a lot, but also have solid evidence, which shows that they have become the mainstream of research now. It is particularly interesting that not all mature topics are equally important: the first kind of topics are more focused on diseases and promoting regeneration, while the second kind of topics are more like springboard to expand research platforms or open new research directions.

Although AF Repair is an obvious big problem in the research field, it is not because people do not pay attention to it-in fact, there are quite a few papers about it. However, the evidence quality of these studies is not enough, and its integration in practical application is also piecemeal. This shows that everyone thinks that AF repair is a key problem in intervertebral disc regeneration, and its success depends on it, but it has not been deeply studied or applied in practice as in other fields. In other words, AF repair is not unnoticed, but there is a big loophole in the research evidence in this field that must be filled.

A third category, called Mechanical Functional Restoration and Composite or Multifunctional Hydrogels, has emerged, both of which are considered as top-notch themes and show strong interdisciplinary integration. Although the original number of these methods is not the highest, they have a great average evidence score, and the recent momentum is very strong, which clearly shows that this field is turning to a more comprehensive strategy, that is, paying more attention to functional recovery, biomechanical repair and multi-faceted design, rather than just focusing on individual materials or biological improvements. These findings mean that the future progress is likely to depend on whether we can focus on the improvement of biomaterials and develop into more complex methods of function and system integration.

When we take a closer look at the thematic maturity reconstruction, we will find that the research on hydrogel-based IVD repair is not just a topic of piling up. Now this field has a finer internal structure, including the mature core direction, the long-term unsolved problems, and the ultra-fast development of new interdisciplinary fields.

### 3.4 Cross-theme comparison and evidence-level heterogeneity

**FIGURE 7:**
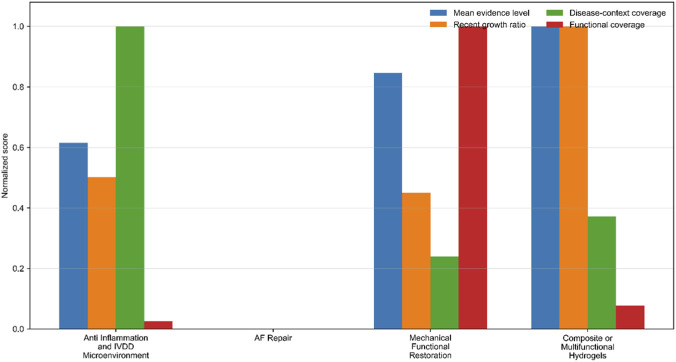
Comparison of key thematic dimensions across representative themes.

In order to better understand the structural differences between our key topics, we analyzed four important aspects: average evidence strength, recent expansion speed, disease range and functional breadth. As shown in Figure 7, Anti-Inflammation and IVDD Microenvironment have the most comprehensive coverage of disease background, which consolidates their position as the most mature disease central themes. On the other hand, the functional scope of AF Repair is obviously limited, which further confirms that it is classified as a structural obstacle. At the same time, mechanical functional restoration and composite or multifunctional hydrogels show stronger integration, and have stronger indicators in terms of evidence depth, recent development momentum or functional focus, which supports their positioning as emerging fields rather than niche marginal topics.

**FIGURE 8:**
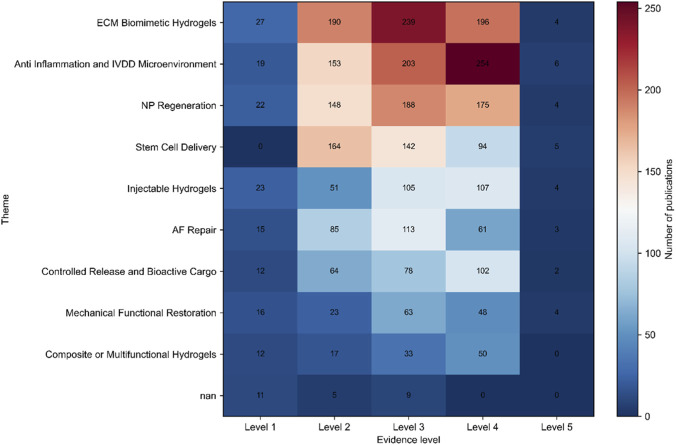
Evidence-level distribution across thematic clusters.

The evidence-level–stratified topic heat map (Figure 8) revealed clear heterogeneity across thematic domains. Mature themes, including ECM Biomimetic Hydrogels and NP Regeneration, were distributed across Levels 2–4, suggesting sustained relevance across multiple stages of research development. In contrast, Anti-Inflammation and IVDD Microenvironment were particularly enriched at Level 4, indicating a recent shift toward integrating degenerative context with disease-mechanistic exploration.

Although Stem Cell Delivery and Injectable Hydrogels are still dominant in the field of moderate evidence level, it is obvious that these mature methods are mainly used as auxiliary platforms, rather than complete disease repair programs, even though they are very powerful in technology and active in research. Similarly, AF Repair focuses on the middle level of evidence, and makes little progress to the higher level of verification-this model explains why this field is still regarded as a bottleneck although many papers have been published. On the other hand, the distribution of mechanical functional restoration and composite or multifunctional hydrogels at a higher level of evidence is more uniform than their overall research scale, indicating that these fields are currently undergoing important method improvement and progress.

Together, these findings show that the situation in the research field of hydrogel-based IVD restoration is actually very different. This field is undergoing an unbalanced change, from focusing on biomaterial in the past to considering degeneration context now, and aiming at functional recovery. This change shows that there are obvious differences between the old research topics, and it is still difficult to solve the problem of insufficient structural repair, while some new strategies are emerging, which need better integration.

## 4 Discussion

In this study, the AI analysis method is used, combined with evidence grade evaluation and theme development analysis, to draw a research map about Hydrogel-based interdisciplinary disc repair. We found several key points. First of all, this field is not only about the study of material properties, but now most of the research focuses on *in vitro*, animal/*ex vivo* experiments, as well as the research related to degeneration. In addition, the recent published articles have not increased evenly in all directions, but have increased sharply with the research of disease-oriented validation, especially those that study the diffusion micro-environment regulation and degeneration-related pathways. Finally, the thematic framework of this field can no longer be measured only by the number of publications, but by the more detailed internal structure-including mature research pillars, long-standing problems, and emerging interdisciplinary directions.

The field has clearly hit its stride, transitioning from the early days of pure biomaterial exploration into a more sophisticated preclinical arena. What’s particularly telling is the prevalence of Level 3 and Level 4 investigations, which signals that scientists are now putting hydrogel systems through their paces in more biologically relevant and disease-specific contexts. This evolution is crucial because the real-world potential of these hydrogels for intervertebral disc repair hinges on more than just their ability to nurture cells or imitate natural tissue—it all comes down to whether they can withstand the harsh conditions of a degenerating disc. The surge in Level 4 studies indicates that the discipline is gradually shifting from simply asking “can this work?” to “how and why does this work?”—a move toward more intelligent, mechanism-driven regenerative strategies.

At the core of this research, there are three very mature methods that focus on disease and regeneration: anti-infection and ivdd microenvironment, ECM Biomimetic Hydrogels, and NP Regeneration. These fields seem to form the conceptual basis of the current research on hydrogel-based IVD repair. Their importance makes perfect sense in biology. The method of microenvironment solves a major obstacle in IVDD pathology, while ECM bionics shows that people have been trying to imitate the natural structure and biochemical characteristics of intervertebral discs. Meanwhile, NP regeneration remains crucial because restoring the nucleus pulposus is key to maintaining disc homeostasis (Chu et al., 2022; Yang X. X. et al., 2023; Li et al., 2026). Simultaneously, several other prominent themes—such as Stem Cell Delivery, Injectable Hydrogels, and Controlled Release and Bioactive Cargo—seemed to function more as facilitative or platform-based approaches rather than fully realized disease-restorative frameworks. This differentiation carries significant weight, given that a high volume of publications should not be mistaken for comparable mechanistic sophistication or clinical applicability.

When we carefully examine all the topics found, AF Repair stands out as the most obvious structural obstacle. This discovery is very important, because AF repair (annulus fibrosus repair) is not a bit part when discussing disc regeneration, but actually a cornerstone in clinical practice and biomechanics (Zhang et al., 2023; Zhou et al., 2024). It is called “bottleneck” because, despite a lot of research, this field is still not good enough in functional integration, and there is less evidence than other topics. On the ground, this means that while everyone agrees AF repair is a must-have, we’re still hitting our heads against a wall when trying to move beyond simple structural fixes to achieve truly durable, functionally integrated solutions that stand the test of time. The hurdles in disc regeneration may stem from the fibrous ring’s anisotropic structure, the uphill battle of restoring tensile properties, the tough job of ensuring seamless fusion with neighboring disc tissue, and the enduring absence of models that can adequately predict long-term structural breakdown and the risk of re-herniation. Consequently, our findings point to annulus fibrosus repair as a critical bottleneck that needs to be overcome before we can hope to achieve truly complete disc restoration.

Although mechanical functional restoration and composite or multifunctional hydrogels may still be relatively small fields, their importance far exceeds their current influence. These research directions imply a major change. The whole field is no longer just adjusting individual components, but begins to adopt a more holistic and systematic thinking. The significance of mechanical functional restoration can’t be overemphasized, because a truly successful intervertebral disc repair requires not only histological improvement or local biological adjustment-it needs to completely restore the mechanical properties of the intervertebral disc, realize the correct load distribution, and maintain functional integrity for a long time. Almost the same is true. The development of Composite and Multifunctional Hydrogels is due to people’s growing understanding that it is far from enough to rely on one material characteristic for the challenging environment of degenerative discs. In the future, the hydrogel system may need to try its best to integrate biomimicry, bioactivity, inflammation management, Mechanical Compensation and even spatiotemporal responsiveness into a unified platform. The rise of these Frontier concepts is also accompanied by the development of the whole field towards more complex and function-driven regenerative approaches.

One of the great advantages of this study is that it uses particularly powerful analytical methods. Common literature analysis methods can find out hot topics, leading countries, famous scholars and important publications well, but they usually can’t tell which ones are superficial and which ones are really deep progress. By using AI-driven evidence classification and topic maturity model, our proposed method has shifted people’s attention from simple research times to more detailed issues: which fields have made the most significant progress, which are not mature enough in concept, and which development directions are most likely to affect the future of this field. Therefore, this framework not only simply describes the situation, but also provides real diagnostic insights. It allows us to regard a large number of documents as a constantly developing knowledge ecosystem, rather than a pile of static and unrelated works.

However, this method also has some limitations that deserve our attention. First, the AI framework relies on domain-specific rule sets and terminology, limiting its generalizability to other tissue engineering fields and failing to fully capture non-standard experimental designs. Second, the analysis only includes 1,085 English publications (2016–2025) from three databases, excluding non-English literature, preprints and gray literature, which may bias the evidence and thematic results. Moreover, although this framework can evaluate the evidence level and subject development in the literature, it cannot directly check the rigor, repeatability or effect of the experiment. Finally, some topics may overlap in biological sense, and multi-topic classification-although necessary for complex regeneration research-may bring some structural duplication. These limitations suggest that the current framework should be viewed as a systematic evidence-mapping tool rather than a replacement for the in-depth evaluation of individual studies.

From a translational research perspective, these findings highlight several key directions for future work. First, annulus fibrosus repair warrants greater attention, as it represents a critical structural and functional challenge—particularly for ensuring long-term tissue integration and preserving normal biomechanical function. Furthermore, the field would benefit from better integration of two strategies: targeting degenerative microenvironments and prioritizing functional restoration, rather than treating them as separate but related objectives. Most importantly, the development of advanced multifunctional hydrogel systems with mechanical restoration capabilities is likely to yield more clinically relevant regenerative outcomes than single-factor optimization approaches. On a broader scale, future efforts should aim to establish more comprehensive platforms that effectively bridge biomaterial design, degenerative pathobiology, and functional recovery.

## 5 Conclusion

In conclusion, this study shows that hydrogel-based IVD repair research is undergoing a structured but uneven transition from biomaterial-centered exploration toward degeneration-context integration and functional restoration. AI-assisted evidence reconstruction revealed that the field is already supported by several mature thematic backbones, but also remains constrained by a major bottleneck in AF repair and a persistent scarcity of high-level translational studies. At the same time, mechanically oriented and multifunctional hydrogel strategies are emerging as the most promising high-integration directions. These findings suggest that the next phase of the field will depend less on further expanding isolated material platforms and more on achieving deeper structural, mechanistic, and functional integration within regenerative disc repair systems.

## Data Availability

The original contributions presented in the study are included in the article/supplementary material, further inquiries can be directed to the corresponding authors.

## References

[B1] Chen S. Wang Y. Wu H. Fang X. Wang C. Wang N. (2023). Research hotspots and trends of microRNAs in intervertebral disc degeneration: a comprehensive bibliometric analysis. J. Orthop. Surg. Res. 18 (1), 302. 10.1186/s13018-023-03788-4 37061725 PMC10105931

[B2] Chu G. Zhang W. Han F. Li K. Liu C. Wei Q. (2022). The role of microenvironment in stem cell-based regeneration of intervertebral disc. Front. Bioeng. Biotechnol. 10, 968862. 10.3389/fbioe.2022.968862 36017350 PMC9395990

[B3] Correa S. Grosskopf A. K. Lopez Hernandez H. Chan D. Yu A. C. Stapleton L. M. (2021). Translational applications of hydrogels. Chem. Rev. 121 (18), 11385–11457. 10.1021/acs.chemrev.0c01177 33938724 PMC8461619

[B4] Dai W. Wu B. Sun J. Shuai W. Han S. (2025). A bibliometric and visual analysis of cancer screening based on the web of science core collection database. Int. J. Health Policy Manag. 14, 8554. 10.34172/ijhpm.8554 41966194 PMC12595571

[B5] Dong S. An S. Saiding Q. Chen Q. Liu B. Kong N. (2025). Therapeutic hydrogels: properties and biomedical applications. Chem. Rev. 125 (18), 8835–8920. 10.1021/acs.chemrev.5c00182 40839830

[B6] Knezevic N. N. Candido K. D. Vlaeyen J. W. S. Van Zundert J. Cohen S. P. (2021). Low back pain. Lancet 398 (10294), 78–92. 10.1016/S0140-6736(21)00733-9 34115979

[B7] Latka K. Kozlowska K. Domisiewicz K. Klepinowski T. Latka D. (2026). Full-endoscopic lumbar spine discectomy: are we finally there? A meta-analysis of its effectiveness against nonmicroscopic discectomy, microdiscectomy and tubular discectomy. Spine J. 26 (3), 479–497. 10.1016/j.spinee.2025.02.006 40024345

[B8] Lee J. S. Lee S. B. Kang K. Y. Oh S. H. Chae D. S. (2025). Review of recent treatment strategies for lumbar disc herniation (LDH) focusing on nonsurgical and regenerative therapies. J. Clin. Med. 14 (4), 1196. 10.3390/jcm14041196 40004728 PMC11856164

[B9] Li X. Zhao Y. Gu P. Zhao C. Fan Y. Zhang X. (2026). Dynamic adaptive coassembled sericin protein orchestrating stem cell development for nucleus pulposus regeneration. Sci. Adv. 12 (1), eadx2768. 10.1126/sciadv.adx2768 41477862 PMC12757073

[B10] Shi J. Bendig D. Vollmar H. C. Rasche P. (2023). Mapping the bibliometrics landscape of AI in medicine: methodological study. J. Med. Internet Res. 25, e45815. 10.2196/45815 38064255 PMC10746970

[B11] Wang N. Rong W. Xie Y. Chen S. Xi Z. Deng R. (2024). Visualizing the bibliometrics of the inflammatory mechanisms in intervertebral disc degeneration. Exp. Gerontol. 188, 112380. 10.1016/j.exger.2024.112380 38382680

[B12] Yang X. X. Yip C. H. Zhao S. Ho Y. P. Chan B. P. (2023a). A bio-inspired nano-material recapitulating the composition, ultra-structure, and function of the glycosaminoglycan-rich extracellular matrix of nucleus pulposus. Biomaterials 293, 121991. 10.1016/j.biomaterials.2022.121991 36586145

[B13] Yang Z. Li H. Lin J. Xing D. Li J. J. Cribbin E. M. (2023b). Research landscape of 3D printing in bone regeneration and bone repair: a bibliometric and visualized analysis from 2012 to 2022. Int. J. Bioprint 9 (4), 737. 10.18063/ijb.737 37323492 PMC10261130

[B14] Zhang K. Feng Q. Fang Z. Gu L. Bian L. (2021). Structurally dynamic hydrogels for biomedical applications: pursuing a fine balance between macroscopic stability and microscopic dynamics. Chem. Rev. 121 (18), 11149–11193. 10.1021/acs.chemrev.1c00071 34189903

[B15] Zhang A. Cheng Z. Chen Y. Shi P. Gan W. Zhang Y. (2023). Emerging tissue engineering strategies for annulus fibrosus therapy. Acta Biomater. 167, 1–15. 10.1016/j.actbio.2023.06.012 37330029

[B16] Zhao Y. Xia Q. Zhu L. Xia J. Xiang S. Mao Q. (2024). Mapping knowledge structure and themes trends of non-surgical treatment in intervertebral disc degeneration. Heliyon 10 (17), e36509. 10.1016/j.heliyon.2024.e36509 39286189 PMC11402762

[B17] Zheng X. Han W. Liu M. Liu L. Ma M. Ye Z. (2025). Incidence, prevalence, and disease burden of low back pain in China: data from the global burden of disease database. Front. Public Health 13, 1563823. 10.3389/fpubh.2025.1563823 40589815 PMC12206742

[B18] Zhou D. Liu H. Zheng Z. Wu D. (2024). Design principles in mechanically adaptable biomaterials for repairing annulus fibrosus rupture: a review. Bioact. Mater 31, 422–439. 10.1016/j.bioactmat.2023.08.012 37692911 PMC10485601

